# Clinical Assessment of Visual Motion Perception in Children With Brain Damage: A Comparison With Base Rates and Control Sample

**DOI:** 10.3389/fnhum.2021.733054

**Published:** 2021-10-07

**Authors:** Ymie J. van der Zee, Peter L. J. Stiers, Lieven Lagae, Heleen M. Evenhuis

**Affiliations:** ^1^Royal Dutch Visio, Rotterdam, Netherlands; ^2^Department of General Practice, Intellectual Disability Medicine, Erasmus MC, Rotterdam, Netherlands; ^3^Department of Neuropsychology and Psychopharmacology, Maastricht University, Maastricht, Netherlands; ^4^Section Pediatric Neurology, Department of Development and Regeneration, University Hospitals KU Leuven, Leuven, Belgium

**Keywords:** motion perception assessment, global motion, motion defined form, motion speed, performance age, PIQ, Performance IQ

## Abstract

**Aim:** In this study, we examined (1) the presence of abnormally low scores (below 10^th^ percentile) in various visual motion perception aspects in children with brain damage, while controlling for their cognitive developmental delay; (2) whether the risk is increased in comparison with the observation and expectation in a healthy control group and healthy population.

**Methods:** Performance levels of 46 children with indications of brain damage (M_age_ = 7y4m, SD = 2y4m) on three visual motion perception aspects (global motion, motion speed, motion-defined form) were evaluated. We used developmental age as entry of a preliminary reference table to classify the patient’s performance levels. Then we compared the percentages of abnormally low scores with percentages expected in the healthy population using estimated base rates and the observed percentages in the control sample (*n* = 119).

**Results:** When using developmental age as reference level, the percentage of low scores on at least one of the three tasks was significantly higher than expected in the healthy population [19/46, 41% (95%CI: 28–56%), *p* = 0.03]. In 15/19 (79% [95%CI: 61–97%] patients only one aspect of motion perception was affected. Four patients performed abnormally low on two out of three tasks, which is also higher than expected (4/46, 8.7%, 95%CI: 2.4–20.8% vs. 2.1%; *z* = 2.61, *p* < 0.01). The observed percentages in the patient group were also higher than found in the control group.

**Interpretation:** There is some evidence that children with early brain damage have an increased risk of isolated and combined motion perception problems, independent of their performance IQ.

## Introduction

Many aspects of a child’s (normal) development, such as emotional, cognitive, social, and physical development, are interconnected. From birth onward, a child starts to integrate different sensory modalities, such as hearing and seeing, to interact with its surroundings. When focussing on visual processing, good visual acuity resolves small details of a retinal image, and the extent of the visual field supports the development and interaction between the oculomotor system and the visual world. Next, brain networks are formed for visual perception, i.e., the ability to recognize and interpret visual aspects of the surrounding environment. During normal development, the development of visual functions depends on the integrity of the eyes and extensive brain networks. If brain damage is present or brain development is hampered, the development of one or more visual functions might be disrupted. The child might be less able to recognize object representations (Vuilleumier et al., 2002; Atkinson et al., 2003; James et al., 2003; Eger et al., 2007), and/or faces (Atkinson et al., 2003), it might have problems with visual attention (Pollmann et al., 2003; Marini and Marzi, 2016), visuomotor integration (James et al., 2003), and/or motion perception (Sunaert et al., 2000; Braddick et al., 2001; Marcar et al., 2004; Stiers et al., 2006; Klaver et al., 2008), even when visual acuity and the visual field developed normally. If one or more of these visual functions are impaired due to brain damage or brain dysfunction a child can be diagnosed with cerebral visual impairment (CVI) and can be considered visually impaired. The presence of (congenital) visual impairment or blindness puts a child’s development of social, communication, cognitive, and motor skills at risk (O’Donnell and Livingston, 1991; Dyck et al., 2004; Brambring, 2007; Houwen et al., 2007, 2008; Tadić et al., 2010) and therefore early intervention and habilitation is considered important.

The importance of motion perception, the ability to recognize and interpret dynamic visual information, is clearly illustrated by the case study of an adult with acquired brain damage (Zihl et al., 1983): LM was unable to visually control the changing fluid level while pouring a drink, to read other persons intentions while they walked through the room and to cross a road. Additional studies in monkeys and human adults suggest that the ability to perceive motion might indeed influence the level of performance on daily activities. A study in monkeys (Born et al., 2000) suggest that the perception of global motion, the segregation of moving objects from the background and fixating and following an object relies on the integrity of the middle temporal area (MT). Global motion perception and smooth pursuit seem essential in humans playing ball sports, i.e., being able to track and predict a ball’s course before catching or hitting it (Land, 2006). Car driving performance seems to be related to multiple motion perception abilities, the performances on a motion-defined form task and a 3D speed discrimination task (Wilkins et al., 2013) and a global motion task (Wood, 2002).

Although these studies suggest a meaningful relation between motion perception abilities and daily activities, motion perception assessment with computerized tasks is currently uncommon in clinical practice. Motion perception tasks with accurate norm values seem not yet available for clinical use. The use of computerized motion perception tasks could have advantages: they not only have quantitative outcomes, i.e., perception thresholds, but also allow to establish the presence and severity of the motion perception impairment. Outcomes of these tasks could be related to performance levels on tasks of daily living. Prior to this, motion perception tasks must be studied in controls and patients, adults and children to set reliable normal limits and to establish whether patient groups are at risk for motion perception deficits.

In the last decades some motion perception studies were done in children. These studies suggested that motion perception deficits are associated with various developmental disorders, e.g., autism, developmental dyslexia, Williams syndrome (WS) and hemiplegia (Gunn et al., 2002; Braddick et al., 2003), Fetal alcohol syndrome (FAS) (Gummel et al., 2012), prematurity (Jakobson et al., 2006) and early brain damage, such as periventricular brain damage (PVL) (Guzzetta et al., 2009). Studies in children with cerebral palsy (CP) or periventricular brain damage (PVL) suggest that various aspects of visual motion perception, i.e., motion-defined form and global motion, can be impaired after early brain damage (Gunn et al., 2002; Jakobson et al., 2006; Guzzetta et al., 2009). Because different studies addressed different aspects of motion perception in isolation, it is currently not known whether various aspects of visual motion perception are impaired in individual children with early brain damage.

Children with congenital or acquired brain damage seem not only at risk for motion perception deficits, but also have lower performance and verbal IQ scores (Bava et al., 2005). Our recent study on the relation between performance IQ (PIQ) and motion perception outcomes in children with brain damage (Van der Zee et al., 2019) showed that non-verbal cognitive intelligence partly explained visual motion perception performance. This means that motion perception scores reflect a patient’s global non-verbal cognitive level, in addition to a possible specific visual motion perception disability. To study the presence of motion perception deficits, one should at least control for a child’s non-verbal cognitive level. Currently, a limited number of studies in motion perception controlled for intellectual disability and/or developmental delay by matching individual patients to individual controls or matching patients and controls on group level (Atkinson et al., 2003; Reiss et al., 2005; Del Viva et al., 2006). In clinical neuropsychological assessment, these methods are not suitable, because of the assessment of individual patients and the common use of sample-based reference tables. To uncover specific motion perception problems, i.e., disentangle general effects of the established non-verbal cognitive impairment from motion perception problems, Stiers et al. (2001) have been suggesting an applicable method: the use of the developmental age (DA), the median age equivalent of (non)verbal intelligence subtests, as entry of the reference table. The lack of control for cognitive delays, the use of the patient’s chronological age (CA) as reference level in neuropsychological assessments might also lead to a profile with more abnormalities and an increase of the number of false positive results (Stiers et al., 2001), resulting in overdiagnosis. The extent of the risk of overdiagnosis is currently unknown.

In this study, we evaluate whether children with brain damage have isolated or multiple motion perception weaknesses by testing three aspects of visual motion perception: global motion, motion speed, motion-defined form. When multiple tasks are used, finding an abnormally low score is less uncommon than in a single task assessment (Crawford et al., 2007). If children with brain damage are at risk for motion perception deficits, then the percentage abnormally low score should be significantly higher than the percentage of abnormally low scores found and/or expected in the healthy population. We studied this in 2 ways: 1. We compared the percentages abnormally low scores (score < 10^th^%) between our control and patient group, 2. We compared the percentage abnormally low scores of the patient group with the estimated base rate of the healthy population.

## Materials and Methods

### Participants

#### Control Group

The control group consisted of 119 typically developing children (54 boys, 65 girls) with no indication of neurological or visual impairments and normal or corrected to normal visual acuity. Controls were recruited through primary schools in the Netherlands (*n* = 79) and Belgium (*n* = 40). At the time of motion perception assessment, their chronological age ranged from 3y6m to 7y10m (*M* = 5y5m, *SD* = 1y0m).

#### Patient Group

To participate in the current study patients had to have (signs of) brain damage, sufficient verbal skills to communicate verbally with the test administrators and a best corrected decimal visual acuity equal to or higher than 0.1 (US notation 20/200 or 1.0 logMAR) to be able to see the dots of the motion perception stimuli. The children were recruited through rehabilitation centers in the Rotterdam area (Rijndam Rehabilitation Centere and Royal Dutch Visio) and the Leuven University Hospital, Belgium.

The patient group consisted of 46 children (23 boys, 23 girls) with indications of brain damage, brain malformation, or clinical indication of visual perceptual impairment. At the time of motion perception assessment, their chronological age ranged from 4y1m to 14y6m (*M* = 7y4m, *SD* = 2y3m).

The studies were approved by the Ethics Committees of the Erasmus Medical Centre (MEC-2006-056) and the Leuven University Hospital. Informed consent was obtained for all participants from their parents or guardians.

### Procedures

#### Medical History and Orthoptic Assessment

Data on gestational age, etiology of the brain damage and imaging results (CT and/or MRI) and recent orthoptic assessments were gathered from available medical records. If no recent orthoptic assessment was done, the child was invited for an orthoptic assessment. Visual acuity with up-to-date refractive corrections (lenses or glasses), visual field, eye movements and binocular vision were assessed by trained professionals (orthoptists). The tests used were matched with the capabilities of the child: e.g., detection visual acuity tests like Landolt C (indicate the open side of the ring) or Teller Acuity Cards (locate the side with stripes) were used in illiterate patients. Visual field was mainly assessed with the confrontation visual field exam (Donder’s test).

#### Developmental Age Assessment

In the current study, the developmental age at the time of IQ-assessment (DA_IQ_) was defined as the median mental age determined by multiple subtasks of a test measuring performance IQ or non-verbal skills (Stiers et al., 2001). This procedure consisted of multiple steps after the standard IQ assessment: 1. Converting the patient’s raw IQ subtest scores to age-equivalents using the appropriate tables in the manual, e.g., you have results on 6 subtests and get the following age-equivalents 54, 57, 43, 52, 83, and 96 months 2. Determining the median of the age-equivalents, the median of the previous example lies between 54 and 57 and is 55.5 months. This is the DA_IQ_. 3. If there was a time-lag between IQ and motion perception assessment, we determined the developmental age at the time of motion perception assessment (DA_mot_) using the follow formula: DA_mot_ = (DA_IQ_/CA_IQ_)^∗^CA_mot_. Note that this procedure can also be used if multiple Performance IQ subtests are done, but no PIQ can be determined.

To minimize the burden on the patients we decided to use recent intelligence results when available. If not available, we only studied non-verbal intelligence, because only non-verbal cognitive ability, and not verbal cognitive skill, is predictive of perceptual performance (Ito et al., 1996, 1997; Stiers et al., 1999). Although the use of a single intelligence test is preferable, the broad age range in the patient group and the cognitive consequences of the brain damage made this impossible. Data of four Intelligence tests were used: the Snijders-Oomen Non-verbal Intelligence Test—Revised (SON-R), the Wechsler Preschool and Primary Scales of intelligence —Revised (WPPSI-R), the Wechsler Intelligence Scale for Children—Revised (WISC-R) and Wechsler Intelligence Scale for Children-III (WISC-III). All four have normative data for the Dutch speaking population of Belgium and the Netherlands. The correlation between SON-R IQ and WPSSI-R PIQ is 0.93 (Moore et al., 1998) and WISC-R PIQ is 0.79 (internal report), the correlation between WISC-R PIQ and WISC-III PIQ is 0.79 (Oosterbaan et al., 2006) therefore we considered these tests interchangeable for the performance age estimation.

#### Motion Perception Assessment

All controls (*n* = 119) and the Dutch patient group (*n* = 17) were tested at the children’s primary schools. The Belgian patient group (*n* = 29) was studied at the Leuven University Hospital. In the Dutch group, motion perception tasks were presented in the order: motion-defined form, global motion, and motion speed. In the Belgian group tasks were administered in random order.

Task administration was done by trained senior psychology students or neuropsychologists. Tasks were presented on a 15-inch CRT monitor attached to a laptop. Participants were placed in front of the screen at approximately 40 cm.

##### Motion Perception Tasks

**FIGURE 1:**
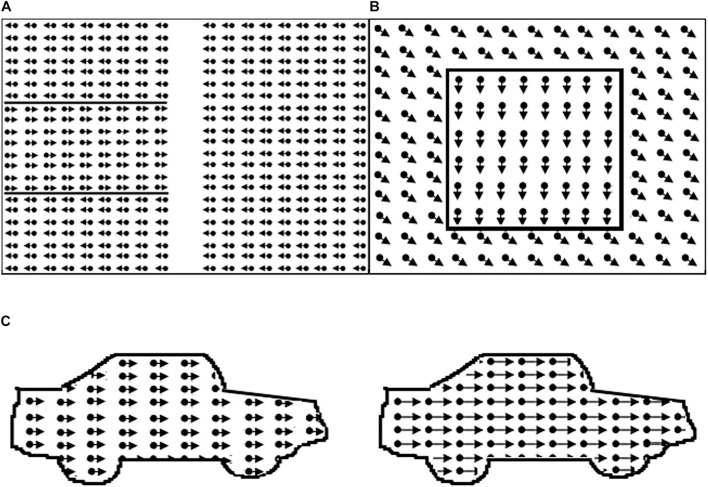
Illustration of motion perception tasks. In the real task borders are not defined by lines. **(A)** Global motion task with target area on the left; **(B)** motion-defined form task, example item square; **(C)** motion speed: dots in right car move faster.

All stimuli (see Figure 1 for examples of the stimuli) consisted of white dots on a black background, with a resolution of 640 × 480 and refresh rate 25 frames/s.

Before each task, example stimuli were used to familiarize participants with task elements and verify that they understood the task.

##### Global Motion (GM)

The global motion stimulus consisted of two random dot kinematograms (size 14.7 × 22.4 deg) containing 1103 white dots (dot size 0.07 deg, limited lifetime 130 ms), presented next to one another with a distance between them (size 3.3 deg). A variable proportion of dots in each kinematogram oscillated coherently in horizontal direction (reversal time 330 ms, velocity 6.7 deg/s). In the middle of one of the random dot kinematograms there was a horizontal strip (size 14.7 × 7.5 deg), where the coherent dots oscillated in the opposite direction. Because the proportion of coherent dots was constant throughout the random dot kinematograms, the strip could not be located by tracing the movement of single dots.

Participants were instructed to help a lost person find his way in the snow by pointing at the strip (maximum stimulus presentation time = 15 s, additional answer time 5 s). A correct answer was followed by a beep. A 2 up–1 down staircase procedure was used (starting level 100%, scaling factor 0.33): i.e., a child had to give two correct answers before the proportion of coherently moving dots, or the coherence level was decreased; One incorrect answer resulted in an increased coherence level in the next trial. After 8 reversals the task ended and the mean of the values of the last 4 reversals was used as the psychophysical threshold.

##### Motion-Defined Form (MDF)

The motion-defined form stimuli consisted of objects hidden in a random dot kinematogram (size 20.6 × 16.0 deg, 5,000 dots, dot size 0.13 deg, lifetime 200 ms, velocity 3.4 deg/s). Each object could be displayed in three successive conditions with decreasing level of difficulty (maximum stimulus presentation time = e. 15 s). In all conditions, the dots outside the contour moved coherently in oblique direction. In the first condition, the dots in the contour of the object moved coherently downwards. In the second condition, the dots in the contour were standing still, and in the third condition there were no dots in the contour. After an object was correctly identified the trial was aborted and the next trial, with a new object, was started. If the object was correctly named or described in the first, second or third condition a score of 1, 0.5 or 0 was noted. If the object was not correctly identified in the third condition the response was marked as inconclusive (INC), and the item was not used in the computation of the visual motion perception score. The motion-defined form score was the mean proportion correct answers on the three tasks. If no score was obtained on one of three tasks, the patient was excluded. Three subtasks, increasing in difficulty, with six objects were presented. Objects in task 1 were: circle, star, bear, banana, heart, and fish; task 2: arrow, kangaroo, boat, guitar, ostrich, and bag; task 3: beetle, seat, airplane, seahorse, car and shoe.

##### Motion Speed (MS)

The motion speed stimulus consisted of two identical contours of a car (car length approx. 17 deg) filled with leftwards moving dots (dot density 11 dots/deg^2^, dot size 0.07 deg, dot lifetime 120 ms). Participants were asked to indicate the location of the fastest car (presentation time 10 s). A correct answer was followed by a beep. A 2up-1down staircase procedure was used (starting speed difference 17.0 deg/s, scaling factor 0.33, 0.25 from fifth reversal). Two correct answers resulted in a decrease in the speed difference of the dots in the cars, which made the task more difficult, an incorrect answer resulted in an increase in speed difference, which made the task easier After 8 reversals the task ended and the mean speed difference of last four reversals was used as the psychophysical threshold.

#### Scoring Task Outcomes

We used preliminary reference data from Van der Zee et al. (2019) to evaluate individual patient performance levels, using their developmental age (if lower than chronological age) as entry for the reference table (Table 1). If the developmental age was lower than 3y6m, task outcomes were compared to results of the youngest reference group. If the age was higher than 7y11m, task outcomes were compared to the results of the oldest reference group.

**TABLE 1:** Reference table, based on a study in 119 typically developing children (Van der Zee et al., 2019).

	Global motion coherence level			Motion speed difference (deg/s)			Motion defined form proportion correct		
	Age in years			Age in years			Age in years		
	3y6m–4y8m	4y9m–5y9m	5y9m–7y10m	3y6m–4y8m	4y9m–5y9m	5y9m–7y10m	3y6m–4y8m	4y9m–5y9m	5y9m–7y10m
*n*	31	39	45	25	34	31	31	43	43
Best	0.19	0.18	0.10	1.85	2.43	0.78	0.93	0.97	1.00
p75	0.34	0.37	0.27	7.14	4.63	2.89	0.78	0.89	0.94
p50	0.46	0.43	0.32	13.74	6.31	4.40	0.64	0.82	0.89
p25	0.69	0.55	0.42	22.53	13.41	7.19	0.50	0.76	0.81
p10	0.78	0.69	0.46	23.80	20.00	12.49	0.45	0.63	0.74
p05	0.80	0.74	0.56	23.80	21.53	19.87	0.33	0.59	0.70
Worst	0.83	0.74	0.64	23.80	22.53	20.96	0.28	0.54	0.64
*95%-CI p*10	2–26	3–25	4–24 *	3–31 *	3–27 *	2–26	2–26	4–25 *	4–25 *

Percentiles for global motion, motion speed and motion defined form task in different age groups. 10th percentile (p10) outcomes were used as cut-off values for abnormally low scores. * 95% CI indicates the precision of the percentiles (range of healthy population that could be excluded using the cut-off values). For this sample, it was calculated for the nearest percentile above p10 giving a whole number of participants excluded.

Scores below the 10^th^ percentile were classified as abnormally low scores or low performance levels.

#### Statistical Analysis

We used a two-stage procedure to estimate whether the performance levels of our patient group were abnormally low. First, we estimated the base rate, i.e.,: the percentage of the healthy population expected to exhibit 1 or more abnormally low test scores (<10^th^ percentile, i.e., *z* = −1.282) on the battery of the 3 motion perception tasks. We calculated the correlations between the motion perception task outcomes in the control group and used these outcomes for the Monte Carlo simulation method described by Crawford et al. (2007) to determine the base rate. Second, we used the estimated base rate as a fixed number and used the binomial test to determine whether the observed percentage of abnormally low scores exceeded the base rate. A one-sided alpha ≤ 0.05 was considered significant.

To determine whether the observed percentage of abnormally low scores in the patient group exceeded the observed percentage of abnormally low scores in the control group we used the independent-samples proportion test. A one-sided alpha ≤ 0.05 was considered significant.

## Results

### Medical History and Orthoptic Assessment

The etiology of brain damage was brain malformation in 3 children, hypoxic-ischemic encephalopathy in 21 cases (18 periventricular leukomalacia, 3 intraventricular hemorrhage), perinatal asphyxia in 5, intracranial hemorrhage in 1, hydrocephalus in 1 and acquired brain injury in 6 (4 trauma, 1 meningitis, 1 tumor). Of the 9 patients in whom no or normal imaging results were present, 3 had a genetic disorder (Velo-Cardio-Facial syndrome; Beckwith Wiedemann syndrome; 46XY + m), 5 had neurological signs such as cerebral palsy, 1 had visual problems not explained by ocular abnormalities and 1 was born dysmature probably due to prenatal drug exposure.

Nineteen out of 46 children (41%) had been born prematurely (gestational age < 37 weeks): 1 extremely premature (gestational age < 28 weeks), 12 very premature (gestational age 28–32 weeks) and 6 moderate to late premature (gestational age 32–37 weeks).

Five patients had ocular abnormalities other than refractive errors or oculomotor dysfunctions, like nystagmus, saccadic dysfunction, convergence abnormality and horizontal oculomotor apraxia. In 22 children, suboptimal or low visual acuity and/or visual field abnormalities were found. Eight had low vision (decimal visual acuity 0.1–0.3, i.e., US notation 20/200–20/63 or 1.0 – 0.5 logMAR) and could therefore be considered visually impaired and 8 children had a subnormal visual acuity for their age (decimal visual acuity 0.5–0.8, i.e., US notation 20/40–20/25 or 0.3–0.1 logMAR). In 7 children a slow or late response was found in one side of the visual field, in 1 child a late response was found in the lower visual field, 2 children had a concentric visual field loss, but one side was more affected then the other and 1 child had a scotoma in the right visual field.

Information on neurodevelopmental and (neuro-) ophthalmologic conditions can also be found in Tables 2 and 3.

**TABLE 2:** Presence of neurodevelopmental conditions in patients with confirmed or suspected brain damage.

Neurodevelopmental conditions	Patient group (*n* = 46)	
	*n*	%
**Etiology**		
Asphyxia	5	11
Hypoxic-ischemic encephalopathy (HIE)		
Periventricular leukomalacia (PVL)	18	39
Intraventricular hemorrhage (IVH)	3	7
Malformation	3	7
Hydrocephalus	1	2
Intracranial hemorrhage (ICH)	1	2
Acquired brain damage (8 months–2.5 years)		
Tumor	1	2
Trauma	4	9
Meningitis	1	2
Genetic	3	7
Unclear	6	13
**Neonatal Condition**		
Prematurity (Gestational age < 37 weeks)	19	41
**Performance IQ (PIQ)**		
Normal IQ (> 84)	11	24
Borderline (71–84)	12	26
Mild retardation (50–70)	10	22
Moderate retardation (< 50)	6	13
Unknown	6	13
**Motor disorder**	**30**	**65**
Spastic cerebral palsy		
Hemiplegia	7	15
Diplegia	7	14
Quadriplegia	3	7
Undefined	1	2
Non-spastic cerebral palsy		
Athetoid	2	4
Ataxic	1	2
Mixed cerebral palsy	2	4
Bipyramidal syndrome	4	9
Developmental delay	3	7

*The bold values are the total numbers beloning to the main category.*

**TABLE 3:** Presence of (neuro-)ophthalmologic conditions in patients with confirmed or suspected brain damage.

(Neuro-)ophthalmologic conditions	Patient group (*n* = 46)	
	*n*	%
**Refractive error**	**9**	**20**
Anisohyperopia	2	4
Myopia	1	2
Hyperopia	2	4
Hyperopia gravior (≥ + 6D)	2	4
Pseudoaphakia	2	4
**Retinopathy of prematurity**		
Stage I or II	2	4
**Optic disc abnormality**	**5**	**11**
Pale appearance	2	4
Smaller than normal	1	2
Optic nerve atrophy (posttraumatic)	2	4
**Strabismus**	**14**	**30**
Manifest	10	20
Intermittent	2	4
Latent	2	4
**Oculomotor dysfunction**	**7**	**14**
Nystagmus		
Manifest	2	4
Latent	1	2
Undefined	1	2
Saccadic dysfunction	2	4
Convergence abnormality	1	2
Horizontal oculomotor apraxia	1	2
**Visual field defect**	**11**	**24**
Scotoma	1	2
Mixed (hemi and altitude)	2	4
Hemianopsia	6	13
Concentric, one side more affected	2	4
**Other ophthalmologic conditions**	**5**	**11**
Bilateral cataract	2	4
Posterior embryotoxon	1	2
Septo-optic dysplasia (SOD)	1	2
Choroidal coloboma + peripheral fundus abnormality + intact optic nerve	1	2

### Developmental Age Assessment

SON-R was used in 17 patients, WPPSI-R in 23 patients, WISC-III in 5 patients and WISC-R in 1 patient. In 6 patients subtasks were done but no Performance IQ was reported. In the remaining 40 patients PIQ ranged from 48 to 121 (*M* = 78, *SD* = 20, *n* = *40*). The available data was sufficient to estimate the developmental age in all patients. DA_IQ_ ranged from 2y4m to 8y1m (*M* = 5y3m, *SD* = 1y5m). The mean time lag between the assessment of non-verbal intelligence and the administration of the motion perception tasks was 2.65 months (*SD* = 3.48). DA_mot_ ranged from 2y5m to 8y2m (*M* = 5y4m, *SD* = 1y5m).

### Outcomes Motion Perception Assessment

To estimate the base rate with the Monte Carlo simulation method (Crawford et al., 2007) we first used the motion perception data of the control group to estimate the correlation between the motion perception tasks. The Pearson correlation between GM and MDF was −0.39, the correlation between GM and MS was 0.22 and the correlation between MDF and MS was −0.42. The estimated percentage of the healthy population that would have 1 or more abnormally low scores was 27.9%. Two or more abnormal scores was expected in 2.1% of the population and 3 abnormal scores in 0.0% of the population.

**FIGURE 2:**
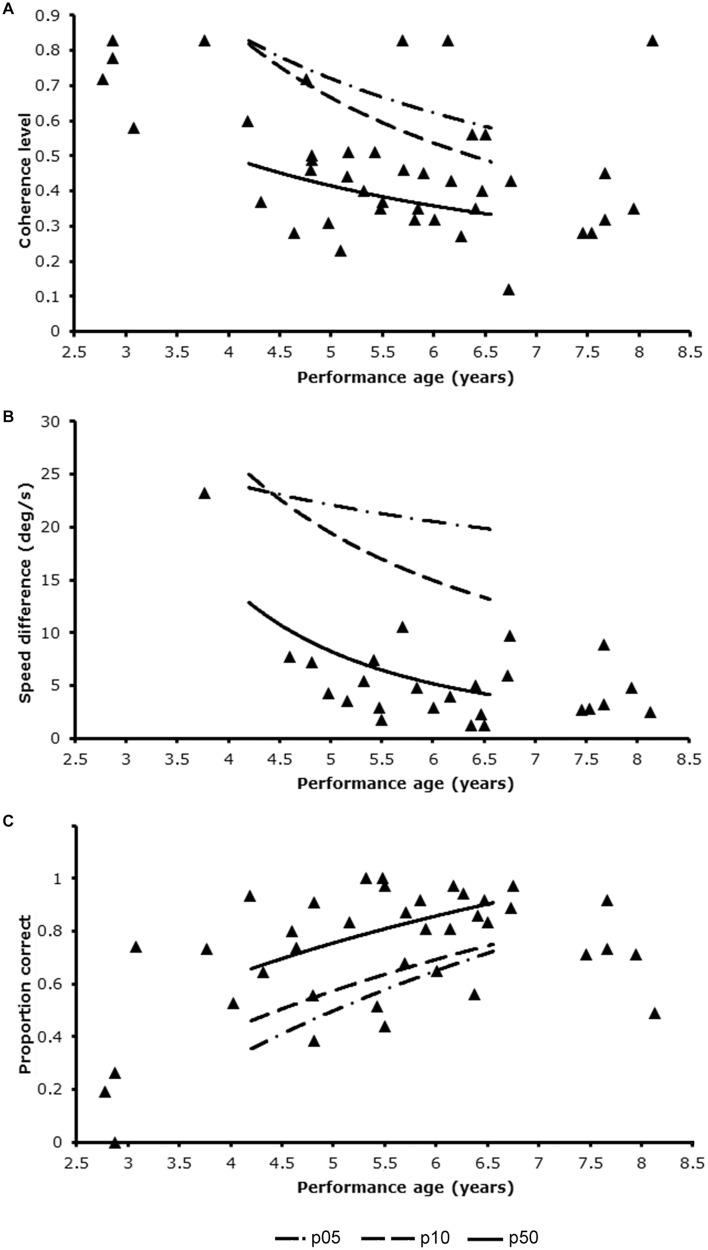
Patients’ performance levels in relation to the trendlines for p05, p10, and p50 of the reference group using developmental age as entry for reference table. **(A)** Global motion task; **(B)** motion speed task; **(C)** motion defined form task.

Of the patient group 8 patients completed 1 task, 17 completed 2 tasks and 21 completed 3 tasks. Figure 2 shows the visual motion perception scores of the patient group relative to the scores in the reference sample. Nineteen out of 46 patients (41.3%, 95%CI: 28.0–55.7%) had an abnormally low score on at least one of three tasks. This was significantly higher than expected in the healthy population (41.3% vs. 27.9%; *z* = *1.88, p* = 0.03). This points toward an increased risk for motion perception problems in the patient group, independent of their performance IQ. Fifteen patients scored below the 10^th^ percentile on a single task. Four patients scored abnormally low on two out of three tasks, which was also higher than expected (4/46, 8.7%, 95%CI: 2.4–20.8% vs. 2.1%; *z* = 2.61, *p* < 0.01).

All 25 patients, which included only 1 Dutch patient, that completed the motion speed task, scored normally on this task (0/25 = 0%, 95%CI: 0.0–13.7%, vs. 10%, *z* = −1.33. *ns*). Ten patients had an abnormally low score on the global motion task (10/42, 23.8%, 95%CI: 12.9–38.1% vs. 10%, *z* = 2.73. *p* < 0.01) and 13 patients on the motion-defined form task, which was significantly higher than expected on a single task (13/38, 34.2%, 95%CI: 19.6–51.4% vs. 10%, *z* = 4.70, *p* < *0.01*).

Mean DA_*mot*_ of the 19 patients with an abnormally low score on any of the tasks was slightly, but not significantly lower than that of patients with normal scores (5y4m ± 1y10 years vs. 5y6m ± 1y1m; *t* = 0.32, *df* = 26.9, *ns*), because it included 4 children with a developmental age under 3 years. Although a developmental age below 3 years did not always result in a low performance level (1 patient scored normally on the global motion task), exclusion of these patients reduced the percentage of abnormally low scores from 10/42 (23.8%) to 7/38 (18.4%, 95%CI: 7.7–34.3%) for the global motion task and from 13/38 (34.2%) to 10/35 (28.6%, 95%CI: 14.6–46.3%) for the motion-defined form task. For global motion the percentage was not different from the expected percentage in the healthy population (18.4% vs. 10%, *z* = 1.46, *ns*). For motion-defined form the percentage was still significantly higher than expected in the healthy population (28.6% vs. 10%, *z* = 3.38, *p* < 0.01).

In the control group 31 of 119 (26.1%, 95%CI: 18.8–34.4%) had an abnormally low score on at least one of three tasks. The independent samples proportion test indicated that the observed percentage in the control group was significantly lower than in the patient group (26.1% vs. 41.3%; *z* = −1.91, one-sided *p* < 0.03). In the control group 3 participants (2.5%, 95%CI: 0.7–6.6%) scored abnormally low on at least 2 tasks. This was significantly lower than in the patient group (2.5% vs. 8.7%, *z* = −1.77, one-sided *p* = 0.04).

Additionally, we explored the characteristics of the patients with abnormally low scores. There was no clear pattern of increased risk for visual motion perception weaknesses associated with any of the etiological categories of brain damage. Low performance levels were found in the following etiological categories: hypoxic-ischemic encephalopathy (6/21, 28.6%, 95%CI: 12.9–49.7%); acquired brain damage (3/6; 50.0%, 95%CI: 16.7–83.3%); genetic (2/3; 66.7%, 95%CI: 17.7–96.1%); unknown (3/6; 50.0%, 95%CI: 16.7–83.3%); other (4/10, 40%, 95%CI: 15.3–69.6%). There was a significant association between low performance levels on the visual motion perception tasks and the presence of low vision (*φ* = 0.33, *p* = 0.03) or the presence of remarkable peripheral visual field outcomes (*φ* = 0.37, *p* = 0.01). It should be noted that these visual conditions were not sufficient to account for the reduced performance on the visual perception tasks. The lowest visual acuity value measured was 0.17 and one patient with this acuity scored normally on the motion defined form task and motion speed task, while another patient with this acuity scored normally on the global motion task.

## Discussion

In this study on motion perception abilities in 46 children with indications of brain damage we assessed whether there is some indication that these group is at risk for motion perception deficits. In current sample we found more abnormally low scores than in our control sample. The observed percentages were also higher than would be expected in the general healthy population. Current results indicate that children with early brain damage have an increased risk for isolated and combined motion perception deficits, while controlling for their non-verbal cognitive level.

Incidences of abnormally low scores (below the 10^th^ percentile) were increased for the global motion task (10/42, 24%) and the motion-defined form task (13/38, 34%), but not for the motion speed task. Importantly, only a small percentage of the patients with impaired motion perception (4/19, 21%, 95%CI: 7.6–42.6%) were impaired on both tasks, indicating that different neural networks are damaged in this patient group. Abnormally low scores were not limited to hypoxic-ischemic encephalopathy, and were also found in other etiological categories of brain damage.

The found incidences were lower than those reported in other studies of neuropediatric populations with comparable cut-off criteria. This difference is most likely due to the more rigorous control for non-verbal performance level in the present study. MacKay et al. (2005), for instance, reported an incidence of 8/19 (42%) of impaired global motion scores in very low birth-weight children with a verbal cognitive ability within the normal range, and age-matched controls. Similarly, in the study by Jakobson et al. (2006), 21/43 (49%) children born preterm and with mild periventricular brain damage scored below 2 standard deviations from the mean in age-matched controls. However, their average performance IQ was almost 20 points lower than that of the controls. It is likely that these incidences, at least in part, also reflected reduced non-verbal cognitive ability.

The use of developmental age as reference level is not only likely to reduce the number of diagnosed problems in groups and individual children, but it will also help neuropsychologists in clinical practice to uncover specific problems and weighing the effect of different neuropsychological factors on the child’s (dis)abilities. Determining and knowing a child’s relative strengths is very important, especially in case of training or support.

The method currently used, the use of the extrapolated median age-equivalent based on PIQ subtests (Stiers et al., 2001), is applicable in clinical practice, even if the IQ-test is not completed. Current method is still quite laborious, another quicker method commonly used in clinical practice is DA = (IQ/100 × CA) (Caplan et al., 2015). *Post hoc* analysis suggested that for the individual patient the way of controlling for cognitive delay matters: we found a mean difference between our DA based on age-equivalents and the DA based on PIQ of 0.7 months (*SD* = 6.2 months) and a range of −16 and 13 months). This means that in several cases a child would be compared to another reference group, possibly with other conclusions.

Another point of attention is that the IQ test used in current study are now outdated. Future studies must make clear whether Perceptual Reasoning subtest performance levels on the WISC-V can also be used to control for cognitive delay. The technical report on the WISC-V gives a correlation 0.74 between WISC-III PIQ and the Perceptual Reasoning Index (PRI) of the WISC-V, which seems reasonable.

It should also be noted that the reliability of the preliminary reference cut-offs is still limited. To set normal limits more precisely, larger samples of typically developing children are needed. Another limitation of our study is that the heterogeneity of the patient population does not allow to delineate specific etiological conditions of risk for visual motion perception disability. Also, evidence for the clinical relevance, i.e., the relation between outcomes of these motion perception tasks and problems in daily life, is still missing. Currently, clinical relevance is primarily based on publication of a single case study, that of patient LM (Zihl et al., 1983). More extensive research on carefully selected patient samples is needed. At this time, motion perception tasks should only be applied as additional observational tools in children with brain damage that have typical problems, for example difficulties in traffic participation.

It is not clear why no weaknesses were observed on the motion speed task. It might be a result of a bias in compliance due to the fixed order of presentation in the Dutch patient group, with motion speed task last: only 1 patient in the Dutch group completed the motion speed task. To avoid this bias in future studies, tasks should be administered in random order. Another possible explanation might be that this was the only task that allowed for a low-level comparison of stimulus features, whereas the other tasks all required a higher level of integration of the moving dots to find the correct answer. Unlike speed discrimination, motion-defined form and global motion required the child to find or identify an object from a limited amount of visual information. This relies on the functioning of more complex networks, the engagement of attention, search and hypothesis testing operations that are associated with frontal-parietal networks (Pollmann et al., 2000; Corbetta and Shulman, 2002). The integrated functioning of posterior visual and anterior executive areas thrives on long-range connection fibers, which may be affected by early brain damage or brain malformation (Ortibus et al., 2012). Additionally, performance levels on the motion speed task might be less dependent on the integrity of primary visual cortex (V1) due to the higher dot speeds used, and might mainly depend on the direct route from the retina to the superior colliculus and pulvinar to the prestriate cortex (Ffytche et al., 1995). The common involvement of V1 in visual acuity, visual field (Duncan and Boynton, 2003) and motion processing (Ffytche et al., 1995) of the global motion and motion-defined form task, might also explain why we found significant correlations between these measures. We suggest, that at least a low-speed task (<6 deg/s) which activates V1 before V5, and a high-speed task (>15 deg/s) which activates the colliculo-prestriate cortical route, should be developed and studied for different motion aspects (Ffytche et al., 1995), in order to study the integrity of different neural networks and the relation with visual acuity and visual field outcomes.

## Data Availability Statement

The raw data supporting the conclusions of this article will be made available by the authors, without undue reservation.

## Ethics Statement

The studies involving human participants were reviewed and approved by the Ethics Committees of the Erasmus Medical Center (MEC-2006-056) and the Leuven University Hospital. Written informed consent to participate in this study was provided by the participants’ legal guardian/next of kin.

## Author Contributions

PS and LL conceived the study, designed the study design and procedures for the study in Belgium. YZ, PS, and HE adjusted the design for the current project, drafted, revised, and prepared the manuscript. PS, LL, and YZ significantly contributed to procedures and execution. YZ and PS interpreted the data. All authors gave final approval for the submitted manuscript.

## Conflict of Interest

The authors declare that the research was conducted in the absence of any commercial or financial relationships that could be construed as a potential conflict of interest.

## Publisher’s Note

All claims expressed in this article are solely those of the authors and do not necessarily represent those of their affiliated organizations, or those of the publisher, the editors and the reviewers. Any product that may be evaluated in this article, or claim that may be made by its manufacturer, is not guaranteed or endorsed by the publisher.
